# Clinical Review, and Hospital Reports

**Published:** 1844-01-01

**Authors:** 


### Clinical Review.

### GUY'S HOSPITAL.

Guy's Hospital Reports. Second Series. No. II. October, 1843. Edited by George Hilaro Barlow, M.A. and M.D. Cantab. Fellow of the Royal College of Physicians, and Physician to Guy's Hospital; Edward Cock, Assistant Surgeon to Guy's Hospital; Edmund L. Birkett, M.B. Cantab. Licentiate of the College of Physicians, and Secretary to the Clinical Society; J. H. Browne, and A. Poland. London: S. Highley.

The Contents of the present Number are as follows:—

1. Observations on Pneumonia, and its Consequences; read before the Physical Society of Guy's Hospital, by Thomas Addison, M.D.—2. Observations on Lithotomy; by Bransby B. Cooper, F.R.S.—3. On the Pathology of Cells; by Thomas Williams, M.B.—4. Case of Extirpation of the Superior Maxillary Bone, performed by William Williams, Esq. of Dolgelly, and reported by him.—5. On a Fæculent Discharge at the Umbilicus, from communication with the Diverticulum Ilei; by T. W. King.—6. On the Extirpation of Ovarian Cysts; by C. Aston Key.—7. Case of Extra-Uterine Fœtation; by B. C. B. Rose, of Swaffham, Norfolk. With a description of the Parts, by Dr. Oldham.—8. Cases of Puerperal Convulsions, with Remarks; by John C. W. Lever, M.D.—9. Report of Cases of Stricture of the Urethra, Retention, and Extravasation, treated in Guy's Hospital from Oct. 1841 to Oct. 1842.

These we shall take up *seriatim.*

#### I. Observations on Pneumonia and its Consequences. By Thos. Addison, M.D.

This Paper is one of too great magnitude and importance to admit of being done justice to here. We shall present a succinct but complete account of it in our next number.

#### II. Observations on Lithotomy. By Bransby B. Cooper, F.R.S.

Mr. Cooper, after some sound preliminary remarks, runs over the symptoms of stone in the bladder, and then passes to the consideration of Lithontriptics, Lithotrity, and Lithotomy.

Of the former, he observes, and we think with justice, notwithstanding what has been said of the Vichy Waters, and so forth—" I appeal to experience as to the result of the experiments which have been made; and believe I may venture to say, that we are not at present possessed of any medicine capable of dissolving, with safety to the patient, any calculus already formed, either in the kidney or bladder." And he adds:—" Nor can much more be said for the efficacy of injections for the cure of stone: they may palliate, but, as yet, we have no evidence of their removing a calculus when once formed; although, like medicines taken internally, they may sometimes relieve the sufferings produced by a stone in the bladder." Such we apprehend to be, paucis verbis, the real state of the case.

Of Lithotrity his opinion is briefly expressed also. It is applicable to cases of small stone, and a bladder capable of containing from eight to ten ounces of water for a considerable time. Nor does "a moderately diseased state of kidney" preclude it. Nay, Mr. Cooper thinks that this might be benefitted by the operation—a problematical point, in our opinion.

Mr. Cooper's directions for the performance of Lithotrity are well worthy of perusal. We quote them, although the passage is a long one:—

"A patient requires but little preparation for the operation of lithotrity; the principal fact to be ascertained is, whether the bladder is capable of retaining a sufficient quantity of fluid to keep it in a fit state of distention to allow the lithotrite to act without fear of injuring the coats of the organ. If the bladder be irritable, the operation should not be immediately proposed; but remedies should be administered to alleviate this symptom, and tepid water should be daily injected into the bladder, increasing the quantity and period of its retention until the patient is capable of retaining eight ounces long enough for the operation to be performed, which may then be considered safe. No further preparatory treatment is required than merely to open the bowels freely before proceeding with the operation. The most convenient position for the patient to be placed, is upon the foot of a low bed, with his feet resting upon two chairs, sufficiently separated as to allow the operator to stand conveniently between them. The bladder should be injected so as to contain about six or eight ounces of tepid water. The lithotrite should then be carefully and gently passed into the bladder, invariably with the screw tightly turned home, so as to secure the close adaptation of the claws of the instrument. The lithotrite is not to be passed into the bladder with the same motions of the hand that the sound or catheter is introduced, but it is rather pushed or pressed onwards, the penis being drawn forwards upon the instrument. Directly the bladder is entered, the screw should be turned so as to release the blades from its influence, and they should be separated: if at this time any water makes its escape by the side of the instrument, an assistant should press the urethra against it, and prevent its flow. The operator then presses the convexity of the end of the lithotrite with considerable firmness against the inferior region of the bladder, so as to render that part the most depending: into this cavity the calculus naturally falls, is immediately felt through the instrument, and is easily grasped by closing the blades; when, by the action of the screw, it is to be broken down, and piece after piece to be seized until it is crushed into fragments sufficiently small to pass through the urethra. As to the number of times the stone may be seized, no definite directions can be given, as it must depend upon the temperament of the patient, and the degree of suffering complained of; but the surgeon may bear in mind, that the more he can safely do at the first operation, or sitting, as it is sometimes called, the better. Each time the lithotrite is passed, the approximation of the blades should be secured by the turning of the screw to its fullest extent; a precaution which is not always taken, but is important, as it prevents the possibility of the blades separating during their passage through the urethra, and the consequent liability of injury to the membrane. The bladder should then be well washed out by injecting it with considerable force, and the water allowed to be drawn off through the catheter as rapidly as it can be made to flow; and in this effort considerable quantity of detritus is sometimes brought away: but this is not invariably the case, for it sometimes happens that but few fragments come away directly after the operation, although the stone may have been effectually crushed. It is not safe to allow the patient to walk or move about after the operation: he should be immediately put to bed, and a large dose of opium administered, to check the liability, which almost invariably occurs, of a rigor, as well as to overcome the irritability of the bladder, excited necessarily by the irritation to which it had been submitted. The prevention of the rigor is a matter of the greatest importance; for if it be not prevented at first, by the opium, there is not only the ill effects of it to overcome, but it seems as if the patient became subject to its recurrence; while, on the contrary, if it be stopped at first the patient seems to be but little prone to it subsequently. This fact I learned from my friend Sir Benjamin Brodie; and am, from experience, thoroughly convinced of the accuracy of his judgment, on this, as on every other surgical subject. For the first two or three days the patient should be kept in bed, and should pass his water in the recumbent posture, not attempting to force the fragments away with his urine until the irritability of the bladder has ceased; and then he should micturate in the erect posture, or even lean forwards, so as to direct the broken portions of stone towards the urethra. If a fragment becomes lodged in the passage, attempts should not be immediately made for its extrication; but a dose of opium should be given, and the patient remain in bed to await the chance of its passing with the next flow of urine. Should this not happen, but still the urine pass, the surgeon should not yet interfere; but if, on the contrary, the urine cannot escape, the fragment must be removed by mechanical means. For this purpose, various instruments have been invented, forceps of different forms; but the best I have seen, and which I have found upon one or two occasions perfectly efficient, is a French instrument, which is made in the form of a straight staff, with a joint at the extreme end and a screw at the top. This instrument is of small size, as it is intended to pass beyond the foreign body in the urethra; the screw is then turned, which acts upon the little joint at the extremity, which is thereby brought to a right angle with the shaft of the staff; and then, by gently withdrawing it, the fragment is drawn up with it. Sometimes, however, these means may fail; and then it will be required to cut the portion of the calculus out of the urethra. This necessity most frequently occurs fortunately at the orifice of the urethra, which is the narrowest part of the canal, and is a matter of no danger as to results. Not so, however, when the stone becomes compacted low down; for although there may be found very little obstacle to its removal, it may prove afterwards very difficult to heal the opening, in which case a fistulous passage may permanently remain. Before, therefore, the surgeon proceeds to cut out a fragment which is compacted low down, every means should be attempted to facilitate its passage with the urine. Opiates, warm-bath, and tartarized antimony, should be exhibited; and should these remedies fail, attempts should be made to push it back into the bladder, that it may be further broken down by the use of the lithotrite. If this attempt fails, then, and not till then, should it be cut down upon, and removed. If, however, it becomes actually necessary to cut down into the perinæum to remove the calculus, I believe the best way of healing the opening, or rather to facilitate its healing, is to use the catheter whenever the patient requires to pass his water for the first week or ten days, so that no urine may pass through the wound; and this plan I think preferable to leaving an instrument constantly in the bladder, as the urine has a tendency to pass between the urethra and the catheter, and so escape through the wound."

Mr. Cooper next turns to *Lithotomy*. He, of course, enjoins a careful examination of the urine, an albuminous condition of it forbidding the operation for the time. He also directs attention to the liver, a fatty degeneration of that viscus being frequent, and lowering the vital powers most unfavourably for an operation.

Mr. Cooper recommends preparatory measures—blood-letting *and* purging, if the patient be plethoric—setting the patient's mind at ease, and, particularly, acquainting him with the position he will have to take, nay inducing him to practise it—a purgative over night, an enema in the morning, with, in irritable habits, a gruel injection with laudanum afterwards.

With regard to the operation itself, Mr. Cooper prefers the straight staff of Mr. Key, " but it must be in the hands of those who appreciate its value and understand how to benefit by the advantages it affords." When this is employed, it cannot, when introduced, be brought to a right angle with an horizontal line, as the curved instrument can, but only to the angle of 45°, beyond which it should not be attempted to be raised, as its point is liable to be drawn out of the bladder.

On the incision in the perineum it is not necessary to dilate. We may observe, however, that, in the second step of the operation, Mr. Cooper opens the membranous part of the urethra, and, then, carrying the knife downwards, as in the first incision, divides the whole length of the deep fascia of the perineum, together with the transverse muscles and artery of the perineum, and some fibres of the accelerator urinæ. The majority of surgeons do not, in this incision, open the urethra, deferring doing so until they intend cutting into the bladder. Mr. Cooper recommends the proceeding he adopts, first, because it offers a precise point for commencing the second incision; and secondly, because it secures the complete division of the deep fascia, a portion of which is otherwise liable to be left undivided, requiring, probably, a further division for the removal of the impediment which it may offer to the extraction of the stone.

In a case in which the artery of the bulb was wounded, and gave rise to a very troublesome hæmorrhage, Mr. Travers ingeniously succeeded in arresting it, by placing a hard compress of cork under his recumbent patient, in such a situation that the weight of his body compressed the internal pudic artery between the compress and the spinous process of the ischium. The precise point where the compress should be placed may be ascertained by drawing a line from the upper part of the trochanter major to the articulation of the os coccygis with the sacrum; and at the junction of the inner with the middle third of this line is situated the spinous process of the ischium, and the internal pudic artery passing over it. The propriety of placing the patient, under these circumstances, upon a hard mattress is sufficiently obvious.

When the straight staff is used a greater difficulty is experienced in opening the membranous part of the urethra, from the greater depth at which it is situated. This Mr. Cooper considers an advantage, from the bulb not being pushed forward, as it is by the curved staff. The artery is thus safer, and the opening into the bladder more direct.

In opening the bladder, Mr. Cooper agrees with the majority of surgeons in deprecating a complete division of the left lobe of the prostate, by which the pelvic fascia is opened, and urinous infiltration rendered too probable. He does not believe that the internal pudic artery is ever, or can be, wounded in the operation for lithotomy, unless it happens to be the subject of some variety in its course, which does sometimes occur—and this, he thinks, might be ascertained by the surgeon during the operation, by feeling with his fore-finger directly after he has divided the deep fascia of the perineum, and consequently before he commences the third step of the operation.

Speaking of the extraction of the stone by the forceps, he observes that a very small one may elude their grasp, and be concealed between their blades. When a small calculus is expected, it is advisable, on this account, to employ flat-bladed forceps. Mr. C. thinks that they are generally made too small, or, at any rate, too short, for adults.

He objects to the knees being tied together, as that tends to direct any blood that may flow into the bladder, where it may accumulate and give rise to urgent symptoms. Mr. C. has frequently witnessed severe rigors a few hours after the operation for the stone, and which have been relieved directly a coagulum has been expelled from the bladder by its own muscular efforts.

Where the calculus is too large to be extracted by the lateral operation, he advises its being crushed by an instrument like the lithotrite, only shorter and stronger, so as to break the stone into two or three pieces, and then remove them with the forceps. The operation *à deux temps* he objects to.

Several operations on the same individual are not very uncommon. Mr. C. has operated three times successfully, on a farmer of Bedford. In the secondary operations, the incisions may be, as before, on the left side of the perinæum.

Mr. Cooper concludes by advising a sufficiently liberal diet *after* the operation. The success of surgeons in this country, when compared with those of the Continent, hinges much upon their attention to this.

### III. On a Fæculent Discharge at the Umbilicus, from Communication with the Diverticulum Ilei. By T. W. King.

It has long been known that occasionally a fæculent or urinary discharge attends the separation of the funis from the new-born infant's abdomen. This discharge usually declines unless connected with some unusual and serious organic derangement. Various cases of irregular and abrupt termination of the bowels may be met with in imperfect fœtuses; and a point of interest in the subjoined pathological account is, that the appearance also depends on a disturbed process of development.

The umbilical vesicle of the human embryo seems analogous to the yolk-sac of the chick in ovo. Here omphalo-mesenteric blood-vessels communicate between the yolk-sac and mesentery; and there is also a trace of a tube, on the plan of a diverticulum, opening into the intestines.

The umbilical vesicle is probably as essential in the first days of the human fœtus as the yolk-sac throughout incubation. Long after the fœtus in utero has established a new source of nutrition and respiration by the placenta, a cord remains, the seeming vestige of a canal from the fading sac (at the end of the funis between the amnion and chorion) to the parts within the abdomen; there is reason to conclude that the site of this canal is that of the intestinal connexion with the common diverticulum ilei, between ten and twenty inches from the cæcum on the convexity of the ileum.

In the Guy's museum there are thirteen, and in that of St. Thomas's Hospital three specimens of diverticulum ilei. The organization and function of these tubes seem closely to resemble those of the bowels. They are from 3 to 10 inches in length. The termination or cul de sac is generally free, mostly rounded. Mr. King sees no more difficulty in explaining the liberation of the diverticular tube from the umbilicus, in the age of active changes and free development, than in the simple atrophy and division of adventitious adhesive cords which we see daily taking place in the adult.

The following is an outline of the cases related.

Case 1.—*Umbilical Fistula.*—John Cashman, æt. four months. Suffered from discharge from the umbilicus, of a thin yellow-colour, and of a faint odour; this discharge had existed from the time the funis came off, on the eleventh day after birth. The aperture of the umbilicus communicated internally with deep sinus (a probe could be passed to the depth of two inches): there was at the time no doubt that it communicated with the small intestine.

Chloride of zinc was first used without benefit; an incision of an ovoid shape was then made around the opening at the umbilicus, and the fresh incised parts brought together by means of three pins and straps of plaster: in a few days the wound was healed, and the patient was discharged cured.

Some little time afterwards the final confirmation of the case presented itself. The little subject appeared to be carried off by internal obstruction and inflammation. A diverticulum, about three inches long, was found adhering to the umbilicus; and an adventitious cord appears to have compressed the ileum, just below its connexion with the diverticulum.

*Case* 2.—A child eight days after birth, presented a fungus occupying the umbilicus, supposed to have been produced by the nurse pulling at the remains of the funis. This fungus was removed, in the course of a few days, by caustic.

The liquid contents of the alimentary canal then commenced oozing through an opening in the umbilicus. An attack of bronchitis supervened; and a portion of intestine, often four inches in length, protruded during the fits of coughing, and its contents might be seen passing through the open extremity. At last, cicatrization was produced; but, about a year afterwards, thoracic disease became manifest, and eventually destroyed the patient.

Upon examining the abdomen, about a foot and a half from the cæcum, a diverticulum nearly five inches long, extended from the convexity of the ileum to the umbilicus, to which the extremity was firmly attached. The umbilicus externally appeared pretty natural, excepting a little central spot of granulation as wide as a pea.

The treatment in these cases should be directed to obviate excoriation, to suppress discharge, to repress undue granulation, or to invigorate the frame, especially if wasted by loss from the bowels. Spontaneous cicatrization concludes the case; although, with neglect, the fistula may remain even to adult life.

### IV. On the Pathology of Cells. By Thomas Williams, M.B.

The discovery by Schleiden and Schwann that all animal and vegetable tissues derived their common beginning from *cells* constitutes a most remarkable æra in the history of Physiology. Notwithstanding, however, the important office, performed by cells in the original formation and subsequent metamorphosis and regeneration of animal and vegetable structures, the author of the present " Report " expresses his surprise that no systematic attempt has hitherto been made towards setting forth the great value and importance of the microscope, as a means for the study and investigation of disease. Pathologists, he says, have been extremely sceptical with respect to the accuracy and fidelity of the microscope, as an instrument of investigation and discovery. Philosophers have always perceived, from Wollaston and Ehrenberg to the least pretending microscopists of the present day, that the endowment and capabilities of the senses, like those of the mind, present palpable individual differences. In examining a morbid structure by the help of a modern achromatic microscope, which enables the observer to resolve the object of study into its *ultimate* and integral *cells* and *filaments*, and *their parent* molecules and capillary system—conducting thus the eye, and therefore the mind, into nearer approach to those more hidden and deeper elements, in the definite characters of which it may read the incipient and secret changes which lead to an accumulation of diseased product, which soon and sensibly proclaims a departure from the healthy state—it becomes at once evident, that this invaluable instrument should in future be considered as the inseparable companion of the morbid anatomist. The difference between a primitive cell and a mass of organised structure, is not simply one of magnitude; for the primitive cell, though not strictly an elementary body, is, in its organisation, infinitely less complex than the mass, and consequently must, under conditions of disease, present changes and phenomena *proportionally* more intelligible than a complex organ. Another point of view in which the dissimilar relations of the primordial cell and the compound organ become peculiarly interesting to the pathologist is, that in animals, as in plants, *all the changes in which organic life essentially consists,* are performed by *cells.* A *cell* is the ultimate limit of organised structure. When the formative blastema assumes the attributes of organisation, a cell is the first visible form under which it presents; it is an atom of organised matter; so that the ultimate cells of organs are the immediate agents of all the *organic processes:* the elaboration of nutrient matter, in all its stages and disintegration, for the purposes of secretion and elimination, are essentially *cell*-phenomena. Hence, it must be obvious that the remarkable advances which these discoveries have effected in physiology, are destined to produce correspondingly important changes in the character of pathological science, and to widen the limits of those narrow bounds within which the routine morbid anatomist has hitherto circumscribed his inquiries. The conclusion is, that the *same* organic laws preside over the combinations and resolutions of the minutest as of the largest aggregations of living matter—a simple primordial *cell,* as of a voluminous compound organ. So true is it, that the most fruitful materials for the successful extension of pathological science lie hidden in the integral elements of structure, by the ponderous mass which progressive changes may have accumulated. If not the *principia,* cells unquestionably are the *semina morbi*—the machinery of propagation. We may now briefly notice the organic laws to which these cells, in common with larger masses, are subjected. A definite scale of development is assigned to the primary organic cells proper to the various structures of the body. These cells pass through prescribed gradations of growth, the duration of their life-period being equally pre-limited. The typical elements of an organic primary cell are three only: first, an external sac (cell-membrane); then a smaller vesicle (nucleus), which contains a smaller (nucleolus). In applying the above description to the *ovum:* the first is the *vitelline capsule* (" membrana vitelli ")—the second the *germinal vesicle* enclosing the third, the *germinal spot.* Primary cells propagate themselves by the reproduction of cells similar to themselves. In its pathological relations this is a very important circumstance; thus, when the malignant tendency has been once established in a part, by the organisation of a *cancerous primary cell,* in virtue of this power inherent in the cell of multiplying its kind, the continuance of the destructive process in the part is certain and inevitable.

Another law with respect to these cells is, that every ultimate vesicle is so far a unity, as that it may be isolated from the surrounding cells, yet depending for its nutrition on the general circulation. Again, analogy leads to the inference that *every cell* is the scene of two descriptions of circulation; one may be styled *diasmotic,** the other *intrinsic,* and strictly nutrient and vital. The nutrient fluid transudes the septum of the *cell*-membrane, and either moves along its inner surface, or combines with its contents—the vitelline structure—to undergo a preparatory elaboration, before it goes to nourish the germinal vesicle. One of two circumstances may produce disease in those delicate organisms, of which one may affect simply the quality of the nutrient circulating fluid, and the other may be associated with some impediment to its circulation. Under the influence of either cause disease will come on the cell. The labour of primary and secondary assimilation devolve exclusively on these ultimate cellules of organic structures. It was formerly supposed that a disordered state of the capillary circulation, called inflammation, was necessary as a prelude to the occurrence of all morbid organic changes. The microscope, however, has introduced important revolutions into the doctrines entertained regarding the essential characters of this process. All the phenomena of which the process consists may be viewed under two divisions; of which the first includes all the changes *mechanical,* chemical, and vital, which the blood undergoes *before* its escape from the capillary vessel; the second, those organising changes, which the plasma (liquor sanguinis) the material which has transuded the parietes of the capillary vessel as a *consequence* of the antecedent condition of the blood *within* the vessels, more or less rapidly puts on. Whatever be the condition, with respect to the chemical and vital composition of the liquor sanguinis, true it is, that from the moment there exists a want of harmony between the diameter of the capillaries and the size of the blood-molecules, obstructions supervene, and *then* appear the characters of inflammation. The entire tendency of the innovations in modern

---

* That is, *perpulsive;* signifying the transit of a fluid through a membranous partition, whether from, or into, a cavity.—*Rev.*

physiology countenance the view, which requires that the process of organization, through the interagency of cells, which takes place in all cases in the inflammatory product, should be regarded as essentially one of nutrition.

*Cells of the Liver.*—The liver should be selected, as the organ affording the best opportunities for prosecuting the interesting subject relating to the *pathology* of cells. The existence of fatty particles within the hepatic cells was determined by Henle and E. Wilson: the discovery of the pathological bearing of this fact is due to Mr. Bowman. He it was who announced the fact, that in one third of the total number of cases of phthisical disorganisation of the lungs, the epithelium cells of the liver become more or less gorged with adipose particles. It is worth mentioning, that no fat globule has yet been found within the *nuclear cell.* It always occupies the interval between the nuclear and outer capsule. With respect to the mechanism of the secerning process, of which these cells are the seat, the situation of the fat seems to sanction the following conclusions:—that the adipose matter is either *secreted*, as a ready-elaborated proximate principle by the cell-membrane; or that the elements of fat, unelaborated, attain in common, and mingled with other principles, the chamber of the *outer cell*, when its elements enter into new combinations: that the *nuclear capsule* is either proof against the entrance of the adipose principles, or that the *nucleus* has not yet attained that degree of development, at which it is endued with the property of secreting fat. If the former be admitted, it must likewise be admitted that the outer cell-capsule possesses the power of *determining the production* of the numerous primitive molecules and the bile-coloured fluid which its chamber contains. During these observations the following positions were arrived at: that the nucleus disappears in the pressure of the increasing fat;—that the primitive molecules, which the hepatic cell normally contains, undergo absorption, according as the adipose particles augment;—that, with the disappearance of the primitive molecules, the *bile-colour* of the cells disappears. During these observations it was ascertained, that in these cells the nucleus could not be viewed as *parentally*, but rather as filially related to the vitelline capsules; that, it was rather the expanding germ of a future generation of cells, than the withered remnant of one passing away; that the nucleus in no way participated in the secerning operation of the developed cell. During the progress of fatty degeneration of the liver, the parenchymal cells, distended by accumulated fat, can no longer sustain their accustomed part in the process of secretion, and they eventually *burst.* Hence, a universal suspension of the secreting agency takes place; so that the last avenue for the elimination of carbon from the blood suffers fatal obstruction, and the lungs and liver becoming then disabled, the whole body is precipitated in the wreck occasioned by the accumulation of carbon in the blood. At first, the undue development of fat in the liver, under the gradual failure of the respiratory agency of the lungs was obviously a protecting alternative; but subsequently the remedy becomes a poison; and the struggling system, now deprived of the agency of two important organs, is soon overwhelmed. Fat contains 80 per cent. of carbon. It must be obvious, that when these two great channels for removing carbon from the system are blocked up by disease, the injurious influence of the retained carbon must be fatal. No one can question the value of these considerations, with respect to the *effects* on the general system, and the pathology of tuberculous disorganisation of the lungs, to which these changes in the liver must conduct.

That the adipose contents of the hepatic cells are destined to constitute a portion of the secretion of the liver, has been determined by the analysis of bile. In *fever*, the adipose globules almost completely disappear from the cells of the liver. In its relation to organic chemistry, no little importance attaches to this fact. This absorption of fat from the hepatic cells in fever, may be viewed as a circumstance depending on conditions exactly the converse of those obtaining in cases of disorganised lungs. The latter organs being reduced to a state more or less completely of disease, the liver becomes burdened with vicarious labour. In *fever*, on the contrary, from the exalted condition of the respiratory agency, the rapid circulation and highly oxidised state of the blood, the decarbonising function of the liver is little required. All the stagnant carbon of the body, thus subjected to the direct chemical agency of a large amount of the free oxygen, with which the blood is impregnated, is pressed into rapid chemical combination, and the products are carbonic acid and water.

Want of space prevents us from extending our analysis of this interesting paper.

### V. Case of Extirpation of the Superior Maxillary Bone. By William Williams, Esq., of Dolgelly.

The patient was a labourer, aged 24. The disease was of two years' duration. The tumor was on the left cheek, of large size, with the skin on its summit red. The nose was turned to the right side by its pressure, and the left nostril completely obstructed: it had also encroached on the orbit, so as to cause a considerable protrusion of the eye-ball. The palate presented a large ulceration, with everted edges; behind which, close to the last molar tooth, was a soft fluctuating swelling: this was punctured, and bled so profusely, that it was with considerable difficulty the hæmorrhage was arrested. The operation was performed Sept. 16, 1841, by Mr. Williams, assisted by his brother, Mr. Rowland Williams, and by Mr. Robert Jones, of Caernarvon.

"The patient was placed on a chair, his head supported and kept steady. An incision was made from the inner angle of the eye straight downwards to the mouth, cutting through the upper lip about midway between the centre and the commissure. A second incision was then commenced from the centre of the zygoma, and carried down to the angle of the mouth, clearing the parotid duct, the integrity of which was preserved: the facial artery bled profusely, and was tied. The flap was now turned upwards, and dissected away from the tumor and the maxillary bone, until the lower margin of the orbit was exposed; after dividing the infra-orbiter artery and nerve, which produced such acute pain, that he fainted, and the operation was suspended until he rallied. Some difficulty was experienced in clearing the edge of the orbit, and in passing the handle of the scalpel between the floor and the eye-ball, as the former had been raised and rendered convex by the pressure of the tumor from below. The buccinator, the masseter, and the temporal fascia were then separated from the superior maxillary and malar bones; while the cartilages of the nose were also detached on the inner side. It now only remained to cut through the connections of the superior maxillary bone; and this was effected with a pair of powerful-bladed forceps. The palatine plate was first divided close to the septum; the zygoma was then cut through; and afterwards the orbiter plate of the malar bone, into the spheno-maxillary fissure. One blade of the forceps was then introduced into the nose, and the other carried along the inner wall of the orbit, by which a division was effected through the nasal, lachrymal, and ethmoïd bones, until it reached the spheno-maxillary fissure. The tumor was then firmly grasped by a pair of forceps, and drawn downwards and forwards: it became loosened from its remaining attachments; and after dividing some fibres, which I conceived belonged to the maxillary vessels and nerves in the spheno-maxillary fossa, the whole mass was brought away. Not more than a pint of blood was lost during the operation, which, together with the dressing, lasted nearly an hour. The parts were brought together with platinum-wire sutures; the cheek was supported internally by dossils of lint, and a bread-and-water poultice applied externally."

In a few weeks the man returned to his work. In July, 1843, he continued in perfect health. The vision of the left eye has improved, but has not been perfectly restored. Muscular motion and sensation are perfect on the left side of the face. The tumor would rather seem to have been fibro-cartilaginous and bony.

### VI. On the Extirpation of Ovarian Cysts. By C. Aston Key.

Mr. Key, although it was long before he could bring himself to regard the extirpation of an ovarian cyst as anything more than a successful accident, now believes that it is an operation simple in its performance, unattended by more than the usual risk of surgical operations, and called for by the hitherto intractable nature of ovarian disease.

Mr. Key argues in favour of its extirpation, and draws a rather forced comparison between encysted dropsy and stone in the bladder. The question turns after all upon this :—what are the dangers and chances of success from the operation ?—and they can be determined only by facts. Mr. Key observes :—

"Entertaining this feeling in favour of the operation, I found, on inquiry, a strong body of experience in the cases that had occurred in the practice of Mr. West, Dr. Lizars, Dr. Clay, Mr. Walne, and Mr. Bird, that sufficiently attested the propriety of the operation ; and the question lay between the choice of the major or the minor incision.

"At the first view, the latter appears to be the safer and easier operation, requiring a smaller excision of the parietes of the abdomen; and exposing, in a much less degree, the viscera to the existing causes of inflammation. These apparent advantages, however, are more than counterbalanced by the difficulty of manipulation which the operator experiences in getting a large collapsed mass through a small parietal incision, and in reaching the peduncle of the cyst so as to secure it by ligature. The larger incision does not probably expose the patient to a greater chance of inflammation than a smaller one; and it has the incalculable advantage of giving free access to the tumor, and facilitating its extraction from the abdominal cavity without violence. I look upon the absence of all undue forcible manipulation as the main recommendation which this operation possesses."

A case presenting itself to Mr. Key, he determined, with the consent of the patient, to operate.

*Case.** E. D., aged 19, a fine-looking healthy girl. The disease had commenced fifteen months previously. On admission, a large tumor, presenting no prominences, occupied the entire cavity of the abdomen. Slight fluctuation felt on the *right side.* On the *left side,* in the lumbar region, a hard mass can be distinctly felt, apparently free from adhesions: it can be traced upwards nearly to the cartilages of the last ribs, and downwards into the pelvis, and seems to be continued into the fluctuating mass on the right side. Position has no effect on its form, nor on the comfort of the patient; nor does pressure produce the smallest uneasiness. The veins are not turgid. The abdomen around the umbilicus measured 49 inches.

The patient was of a nervous and highly excitable temperament. The nature of the case, and the risks of operation were explained to the patient and her friends—their assent to its performance was obtained—a proper room was provided, and the girl removed to it—and at 3, p. m., on the 1st of August, the operation was performed in the following way, by Mr. Key, the temperature of the chamber being previously heated to 70° Fahr.

"Mr. K. commenced the operation by making an incision, about four inches

---

* We trust the *cases* in these Reports will be related a little less in detail. Their prolixity out-Herods Herod, and, instead of instructing, fatigues.

long, below the umbilicus, in the median line; and, after carefully dividing the several layers, exposed the peritoneum. A small opening was made into this, and enlarged upon a director: several ounces of dirty-coloured ascitic fluid escaped. Mr. Key then passed his finger through the wound, on either side of the tumor, and found it perfectly free. An incision was now made from near the ensiform cartilage to the upper extremity of the first incision: the integuments and superficial fascia were cut through by this sweep of the knife, and the peritoneal cavity freely laid open to the same extent by a broad probe-pointed bistoury, carried from below upwards upon the finger: the lower extremity of the first incision was also enlarged by means of the bistoury. The tumor was thus perfectly exposed, and presented no adhesions whatever: there was little or no bleeding from the superficial vessels: the abdominal parietes were thicker than might have been expected. The edges of the wound were kept in close contact with the surface of the tumor, which Mr. Key allowed to escape gradually, assisting its protrusion by depressing the sides of the parietes, which was requisite on account of the great size of the cyst in its antero-posterior diameter. There was a cord-like peduncle at the right side of the lower part of the tumor, around which a single ligature was placed and divided. The tumor, at it issued from the abdomen, was received by Dr. Lever, and gently raised; while the peritoneal cavity was immediately closed behind it, by approximating the edges of the incision. Much ascitic fluid escaped, which required carefully sponging up. Mr. Key now passed a strong needle, fixed to a moveable handle, and armed with a double ligature of strong silk, through the centre of the peduncle. The lower ligature was tied pretty easily; but the upper one required a great deal of force, owing to the broad expansion of the ligament. The peduncle was now divided between the ligature and the tumor, and the diseased mass at once lifted away: there was not the least bleeding. The three ligatures were placed at the lower extremity of the incision, and the edges of the parietes brought together, and retained in juxta-position by means of about twelve interrupted sutures. The operation did not last long, and the patient bore it extremely well. Long strips of adhesive plaister were placed at the interruptions of the sutures, and served to keep the edges of the wound in close contact; a broad eight-tailed bandage, which had been laid ready under the loins and back of the patient before the operation, was now uniformly applied, and the girl placed in bed."

She died at 8, A.M., of the 5th of August, having had more or less rapidity of pulse, disposition to vomiting, slight shivering, and, latterly, low delirium.

There were evidences, on dissection, of low inflammation of the peritoneum, especially about the pelvic cavity.

### VII. Cases of Puerperal Convulsions. With Remarks. By John C. W. Lever.

Mr. Lever is very favorably known to the profession by his zeal in the prosecution of obstetrical pursuits, and many valuable contributions to their literature. In the paper before us, he details the particulars of fourteen cases of puerperal convulsions. These have occurred between the years 1834 and 1843, out of seven thousand four hundred and four women attended by the pupils attached to the Lying-in-Charity of Guy's Hospital. The main feature which has struck him has been the coincidence of an albuminous condition of the urine, in nine out of ten cases in which that secretion was examined. The following heads of the cases will give an insight into their general characters, and their results, more seeming unnecessary, as the symptoms and treatment present nothing new nor extraordinary.

*Case* 1.—Fifth Confinement—Anæmic Convulsion from Loss of Blood—Mother recovered—Child born alive.

*Case* 2.—Primipara—Unmarried—Mother recovered—Child born alive.

*Case* 3.—Primipara—Anæmic Convulsions—Child born alive—Mother recovered.

*Case* 4.—Convulsions before delivery—9th Confinement—Mother recovered—Child born alive.

*Case* 5.—Primipara—Version—Urine highly albuminous—Mother recovered—Child still-born.

*Case* 6.—Anæmic Convulsions—Married—4th Confinement—Partial Placental Presentation—Mother recovered—Child still-born.

*Case* 7.—Primipara—Unmarried—Child expelled by the natural efforts—Urine coagulable—Mother recovered—Child born alive.

*Case* 8—Primipara; delivered by the Forceps—Urine albuminous—Child born alive—Mother recovered.

*Case* 9.—Second Confinement—Convulsions after Delivery—Urine Albuminous—Mother recovered—Child born alive.

*Case* 10.—Ninth Pregnancy—Child Still-born—Mother died—Inflammation of the Membranes of the Brain.

*Case* 11.—Primipara Convulsions—Delivered by Forceps—Emphysema—Death—Child alive—Urine albuminous.

*Case* 12.—Unmarried—Primipara—Convulsions supervening after delivery—Child living—Mother recovered—Urine albuminous.

*Case* 13. — Primipara — Convulsions — Twins — Forceps — Turning — Urine albuminous—Mother recovered—Children alive.

*Case* 14.—Second Confinement—Forceps—Mother recovered—Child born dead.

From the consideration of these and of other recorded cases, Dr. Lever deduces several, not unimportant, statistical conclusions.

*Ratio of Mortality.* (1, *to mother*). Out of fourteen cases related here, two only were fatal. The mortality has been ranked much higher. Dr. Lever collates 166 reported cases. In these, 44 women died, or 26·5 per cent. If the fourteen cases recorded in this Paper be omitted, the number of cases will be 152, and the number of deaths 42, or 27·6 per cent.; while the cases attended at the Lying-in-Charity of Guy's Hospital have proved fatal only in the proportion of one in seven, or 14·2 per cent.

(2. *To Children.*) The fourteen women gave birth to fifteen children; eleven of whom were born alive, and four still-born. Of the four still-born, two were delivered by the operation of turning; one by the employment of the forceps; and the other was a case of partial presentation of the placenta, in which the child descended with the feet forwards.

*Cases of Labour and Method of Delivery.*—In seven cases the children were born by the natural efforts; in three, the forceps were employed; in two, the operation of turning was resorted to: in one case there were twins, the first of whom was delivered by the forceps, the second by the operation of version; and in the last there was a partial presentation of the placenta.

Nine of the women were married, and five unmarried. Of the fourteen women, eight were primiparæ, and six had been previously confined.

It appears that primiparæ are most obnoxious to convulsions.

In two of the cases, convulsions supervened before labour was established; in ten, they occurred during the progress of the labour; and in two, they did not exhibit themselves until after the birth of the child and the expulsion of the secundines.

*Condition of the Urine.*—Dr. Lever would seem to be the first who has drawn attention to the important fact that, in puerperal convulsions the urine is prone to be albuminous. But *in no cases of pregnancy has he discovered albuminous urine, save in those in which there have been convulsions, or in which symptoms precursory of puerperal fits have presented themselves.* The combination of

œdema of the face and extremities with convulsions has been remarked by several obstetrical writers. Dr. Lever adds :—

"From what I have seen in public and private practice, I am led to the conclusion that cases of convulsions complicated with an albuminous condition of the urine are divisible into two forms; in the one, the urine is *albuminous during pregnancy;* and there are external evidences, as shewn in the œdema of the face, eyelids, hands, &c. In such cases, the convulsions will be more violent, and will last for a longer time after delivery. The urine also retains its albuminous properties for a longer period than in the second form, or that in which the urine becomes *albuminous during the labour.* In this variety, the urine contains less albumen; the fits are less violent; seldom re-appear after delivery has been completed; and if they do, it is in a milder form, unless complicated with some lesion of the brain. The urine, in this form, very speedily loses all traces of albumen after labour is completed. Mr. Robinson in his Monograph, has satisfactorily proved, that causes which induce congestion of the kidney by preventing or obstructing the return of blood through its veins, as abdominal tumors, &c., will produce renal congestion and albuminous urine: and I am of opinion, that the gravid condition of the uterus, by its pressure, prevents the return of the blood through the emulgent veins; and hence is the cause of the renal congestion, and the consequent albuminous condition of the urine. This opinion is supported by the facts I have already adverted to; viz. that the urine was found to be albuminous only in those women who were affected by, or who had the premonitory symptoms of convulsions. The pressure of the gravid uterus is by no means uniform, as stated by some writers: this may be remarked in the difference in size and figure that females present in their several pregnancies. In some, the uterus is distended more at its posterior and lateral parts than at its anterior, and *vice versâ;* and yet, in both, the contents of the uterus may be equally great.

"In these cases, the congestion will take place and the urine become albuminous towards the close of pregnancy: but this untoward pressure may not be excited until the onset of labour; and as this progresses, the congestion may be increased, and consequently the albuminous condition of the urine be caused. Thus, in my opinion, we have the same cause producing this condition of the urine both during pregnancy and parturition. The great similitude that exists in the appearances presented by females attacked with eclampsia, and those observed in persons affected with albuminaria, must have been oftentimes noticed by those who had attentively regarded both."

*The blood* was analyzed in one case, without detecting urea.

*Treatment.*—The cases are divisible into two kinds—anæmic and sthenic. The objects in the latter are to cut short the paroxysm, remove the coma, and guard against another fit—objects to be attained by relieving the vascular excitement and congestion, and putting an end to the congestion.

Active depletion—tartar emetic, to nausea, sometimes combined with a small quantity of opium to prevent irritation of the intestinal mucous membrane—purgatives, and where deglutition is imperfect or lost, calomel in butter on the tongue is recommended—mercury, regard being had to the albuminous urine, with caution. Delivery, as soon as is consistent with the state of the patient herself and the condition of the parts through which the child has to pass—such are the remedies advised by Dr. Lever. He adds :—

"And thus, while, on the one hand, I deprecate artificial dilatation of the os uteri (which may induce a convulsion)—while I am no advocate for rupturing the membranes, and the induction of premature labour—while I declare myself altogether opposed to incisions in the vaginal portion of the os uteri, as recommended by Velpeau—I do most strongly recommend that delivery be resorted to so soon as the state of the parts will permit its accomplishment.

"If the membranes be unbroken—if the os uteri be soft and dilatable—if the external parts be lax and moist—then version may be readily performed: but unless there are circumstances of great moment, calling for the immediate delivery of the woman, I would rather wait until the head of the child can be grasped by the forceps or vectis, and the delivery be, by their aid, accomplished."

---

### ST. GEORGE'S HOSPITAL.

### REPORT OF SOME CASES OCCURRING IN THE PRACTICE OF MR. HENRY JAMES JOHNSON, ASSISTANT-SURGEON TO THE HOSPITAL.

#### I. ULCERS OF THE LEG.

In the Number, before the last, of this Journal, there appeared a brief Report on the Management of Ulcers of the Leg. The object of it was to recommend the application to the sores of slightly stimulating lotions, after the manner of water-dressing, in lieu of that of ointments. Some cases were detailed with the view of shewing the good effects of the plan, which has since been acted on with satisfactory results. I would now take the liberty of offering an additional remark or two.

When the veins are varicose, as they commonly are, no applications can be expected to succeed, without the assistance of some description of bandage. The latter, however, is useful and necessary, when no enlargement of the veins exists, the uniform temperature and support that it ensures, contributing to the healthy actions of the sore. But an ordinary roller is not found to answer so well as strapping, which makes more equable pressure, is less apt to be deranged, and needs less frequent renewal.

The usual mode of strapping a limb, where one strip of plaister overlies the other, in an uninterrupted series, is incompatible with the proper application of liquid dressings. Under such circumstances, *they* are either suffered to remain too long unchanged—or such trouble is incurred with the strips of plaister, as to induce neglect, and render the whole plan nugatory. Finding this to be the case, we have latterly pursued the following method:—

Strips of Soap Plaister are applied, in the usual way, to the limb, up to the level of the sore. Above this, strips are applied also, and oblique, or cross strips connect the two, the ulcer, with a little of the skin around, being left uncovered by the plaister. If several sores exist, the strapping is modified accordingly—when they are confluent, or contiguous, the aggregate being left exposed—when separate, the strips being carefully carried between them. In this manner, the limb gets adequate support, while the ulcers are open to as frequent a renewal of the liquid dressing as is necessary. Our success has been such as to induce me to recommend this mode of proceeding very strongly.

A not unfrequent combination is that of acute, or chronic, eczema, with varicose veins and ulcers of the leg. We see this so often as to render it probable that the varicose veins are, at least, a predisposing cause of the Eczema. The case is troublesome, especially when the eruption is chronic. The irritability of the skin forbids the application of adhesive, or of soap strapping, while the state of the veins demands compression and support. What I have found to answer best, is to apply strips of calico, on which has been spread the compound chalk-ointment, or that of zinc and lead, after the fashion of soap strapping, leaving the sores free for the liquid dressing. But attention is requisite on the patient's part, for the ointment should be renewed once, at all events, in the course of the twenty-four hours, and the greatest cleanliness should be observed.

#### II. ECZEMA.

This may be said to be our staple Eruption. We see it at all ages, but more particularly in the young and old—in all parts of the body, but most in the head and on the lower limbs—and with every shade of intensity, but more frequently subacute and chronic than acute. If I may trust my own experience, in private practice, the same thing obtains with the better classes of society, and Eczema is the eruption most rife in all.

The descriptions of the complaint contained in the works of Rayer, Cazenave, or Wilson, are too popular and too complete to require or admit of many additions. The ensuing remarks are no more than a few hints, drawn from my own observation.

In the first place, I would observe, that, either the eruptions of the scalp in children have greatly altered, of late years, or their nature was misunderstood. Hospitals were formerly crowded with specimens of intractable scalp disease, for which the severest remedies were employed in vain. Now we see little of the sort. In the course of nearly a twelvemonth, I have had but three cases of Favus, (one of those was dubious) and all were cured in a few weeks. But Eczema, and Eczema Impetiginodes of the scalp, have been of daily occurrence, and I have little doubt that, had irritating applications been resorted to, we should not long have been without inveterate cases.

In the next place, it appears to me that Eczema is one of those diseases, which may be adduced in favour of a rational Humoral Pathology. Whether we look at the circumstances of its occurrence, or the effects of treatment on it, we must equally be struck with the evidence it offers, of a vicious condition of the blood. We find it in the cachectic child, fed on insufficient and improper nutriment, and in the luxurious or indolent members of the upper classes, accustomed to too high a scale of diet. The remedies that control it are such as would modify the nature of fluids—evacuants of every kind, purgative, diuretic, sudorific.

I would not be understood to deny, or underrate, the influence of local causes. We find a marked tendency to it on the hands, especially in those whose occupations expose those parts of the body to changes of temperature or sources of irritation. It is very common in bakers. I have already alluded to its frequency upon the legs, in connexion with a varicose state of the veins, the imperfection of the cutaneous circulation appearing, in this instance, to dispose to it. On the scalp, it is so rife in the children of the poor, as to make it not unlikely that want of cleanliness operates materially in its production.

It will be found in many instances, although there are numerous exceptions to the rule, that the subjects of Eczema, especially adults, have a naturally dry state of skin. This probably acts in two ways. The deficiency of the unctuous secretion exposes the integument to suffer more from cold or any irritating agents, while the faulty transpiration does not rid the blood of the lactic or acetic acid, and the other principles which the skin is intended to excrete. It is right, however, to observe, that a dry skin is more prone to Psoriasis than Eczema.

At another opportunity, I shall take the liberty of reverting to this subject, and shall content myself at present with a few observations on the Treatment.

I would say that, on the whole, there are few complaints, of the same severity, more controllable than Eczema. In the majority of instances, it is immediately relieved, and it is not slow of being cured. This applies to cases of moderate duration, and which have not been aggravated by improper management. When the disease has existed long—has been irritated by stimulating applications—or attends on a varicose state of the veins, it will, probably, be tedious, and *may* be irremediable.

The two great objects in the treatment of Eczema, are to restore the healthy state of the blood and the secretions, and to calm local excitement. Evacuants and sedative applications are the means for fulfilling these intentions.

The blue-pill with ipecacuan, at night, followed by morning doses of senna and sulphate of magnesia—or the combination of the sulphate and carbonate of magnesia with colchicum and gentian—full doses of the liquor potassæ in toastwater, or in barley-water—regular warm baths—ensure free discharges from the bowels, the kidneys, and the skin. If the patient be strong, we must not hesitate to carry the purging and the other evacuations to a decidedly lowering extent. In acute Eczema, and a full habit, we may commence with venesection. But purging, active purging, seems to be the key to successful practice.

The part should be bathed twice or thrice daily with thin, warm gruel or bran tea, and an ointment composed of equal parts of the Unguentum Zinci and Ceratum Plumbi, should be kept constantly applied; the combination of chalk and lead answers, but not so well as the preceding.

When the reduction of the patient has been carried far enough, and the disease is subsiding, the purgation should be moderated, and sarsaparilla may be given with the liquor potassæ. This serves as a mild tonic, and accelerates the cure.

As a general rule, the patient should refrain from all fermented liquors, take little animal food, and avoid all sweets and acids.

The gist of the treatment, then, consists in this—to reduce the patient to a certain point, by purging, alkalies, and diuretics; and, when the vascular excitement of the part is subdued, to give sarsaparilla, or some mild tonic, along with liquor potassæ, maintaining the action of the skin by baths, and that of the bowels by moderate aperients. The part itself is tranquillized, throughout, by warm ablutions, and by the sedative influence of lead, in combination with chalk or zinc.

Of course, the more acute the Eczema, and the more robust the patient, the more active would be the depletory measures. But when the cachectic are the subjects of the complaint, we must be more chary of purgation, and be cautious not to lower the system too much. In cases of this kind, in adults, a mixture, containing the sulphate and carbonate of magnesia, with infusion of hop or gentian, answers the purpose very well. In some instances, I have seen good effects from sulphur in connexion with nitre and soda,* and it seems to suit children especially. The hyd. c. cretâ, with rhubarb at night, and castor-oil, or manna, in the morning, are also adapted, as aperients, for this class of patients. It is unnecessary, however, to particularise such modifications of remedies as will suggest themselves to every experienced person.

I am inclined to think, that whatever benefit the liquor potassæ may exert as a pure alkali, (and, as such, it must influence the constitution of the fluids,) no trivial part of its curative action is that which it effects through the kidney. I fancy that in some, at least, of the cases in which I have seen this medicine of most service, there has been a marked diuresis. That it is a lowering agent is certain, the patients being usually more or less blanched, and attenuated by it, when taken in full doses, or for any time. In accordance with this double view of its operation, I prescribe it in the early stage of the treatment, in a large quantity of simple diluent, such as barley or toast-water. At a later period, sarsaparilla, or some bitter infusion, is combined with it, to counteract undue depression of the system. Well directed, it is in this, and in many disorders, a drug of extreme value.

In conclusion, I would caution medical men, not to be over-anxious to cure Eczema, in elderly persons who have had it long. That is not to be done with safety. The system, in them, is accustomed to the local determination and congestion, and if such be barred from one quarter, it will probably seek another. The head is the part that will suffer. Of this I have witnessed some striking instances.

8, *Suffolk Place, Pall Mall.*

---

* This remedy is, I believe, a favourite one with Mr. Cæsar Hawkins. The formula is—Sulphuris loti ʒj, Potassæ nitratis gr. v, Sodæ bicarb. gr. vj; bis vel ter quotidie.

